# Prevalence of protective tetanus antibodies and immunological response following tetanus toxoid vaccination among men seeking medical circumcision services in Uganda

**DOI:** 10.1371/journal.pone.0209167

**Published:** 2018-12-31

**Authors:** Fredrick Makumbi, John Byabagambi, Richard Muwanika, Godfrey Kigozi, Ronald Gray, Moses Galukande, Bernard Bagaya, Darix Ssebagala, Esther Karamagi, Mirwais Rahimzai, Mugagga Kaggwa, Stephen Watya, Anthony K. Mbonye, Jane Ruth Aceng, Joshua Musinguzi, Valerian Kiggundu, Emmanuel Njeuhmeli, Barbara Nanteza

**Affiliations:** 1 Makerere University, College of Health Sciences, School of Public Health, Kampala, Uganda; 2 Rakai Health Sciences Program, Kalisizo, Uganda; 3 University Research Co., LLC (URC), USAID Applying Science to Strengthen and Improve Systems (ASSIST) Project, Kampala, Uganda; 4 The Johns Hopkins Bloomberg School of Public Health, Baltimore, MD, United States of America; 5 Makerere University, College of Health Sciences, School of Medicine, Kampala, Uganda; 6 Makerere University, College of Health Sciences, School of Biomedical Sciences Kampala, Uganda; 7 World Health Organization, Uganda Country Office, Kampala, Uganda; 8 UroCare Hospital, Kampala, Uganda; 9 Ministry of Health, Kampala, Uganda; 10 Office of HIV/AIDS, Global Health Bureau, United States Agency for International Development (USAID), Washington, DC, United States of America; Institut Pasteur, FRANCE

## Abstract

**Introduction:**

Tetanus infection associated with men who had male circumcision has been reported in East Africa, suggesting a need for tetanus toxoid-containing vaccines (TTCV).

**Objective:**

To determine the prevalence of tetanus toxoid antibodies following vaccination among men seeking circumcision.

**Methods:**

We enrolled 620 consenting men who completed a questionnaire and received TTCV at enrollment (day 0) prior to circumcision on day 28. Blood samples were obtained at day 0 from all enrollees and on days 14, 28 and 42 from a random sample of 237 participants. Tetanus toxoid (TT) IgG antibody levels were assayed using EUROIMMUN. Analyses included prevalence of TT antibodies at enrollment and used a mixed effects model to determine the immunological response.

**Results:**

Mean age was 21.4 years, 65.2% had knowledge of tetanus, 56.6% knew how tetanus was contracted, 22.8% reported ever receipt of TTCV, and 16.8% had current/recently healed wounds. Insufficient tetanus immunity was 57.1% at enrollment, 7.2% at day 14, 3.8% at day 28, and 0% at day 42. Antibody concentration was 0.44IU/ml (CI 0.35–0.53) on day 0, 3.86IU/ml (CI 3.60–4.11) on day 14, 4.05IU/ml (CI 3.81–4.29) on day 28, and 4.48IU/ml (CI 4.28–4.68) on day 42. TT antibodies increased by 0.24IU/ml (CI 0.23, 0.26) between days 0 and 14 and by 0.023IU/ml (CI 0.015, 0.031) between days 14 and 42 days. Immunological response was poorer in HIV-infected clients and men aged 35+ years.

**Conclusion:**

Insufficient immunity was common prior to TTCV, and a protective immunological response was achieved by day 14. Circumcision may safely be provided 14 days after vaccination in HIV-uninfected men aged less than 35 years.

## Background

Uganda adopted a Safe Male Circumcision (SMC) policy in 2010, following three randomized clinical trials showing efficacy for HIV prevention in men [1–3]. The prevalence of HIV among adults aged 15 to 64 in Uganda remains high at 6.2%: 7.6% among females and 4.7% among males, and 0.4% among 0–14 years [4].

Ministry of Health (MoH) policy recommends SMC for all ages, particularly males aged 10–49 years. The funding for the program is mainly from the U.S. President’s Emergency Plan for AIDS Relief (PEPFAR), with services provided by implementing partners (IPs). Over the past six years, nearly 3.2 million circumcisions have been performed in Uganda [5] using conventional surgical methods (95%), with a much smaller number circumcised using the PrePex elastic collar device for adult men and the Mogen clamp for infants. Use of other devices has been piloted in Uganda [6–7]. Seventy percent of all the circumcisions are conducted in mobile SMC camps.

Cases of tetanus infection following circumcision have been documented in West Africa [8] and in East African countries, including Uganda [9]. Post-circumcision tetanus infection may be acquired through the circumcision wound or other lesions, providing an anaerobic environment for the growth of Clostridium tetani. The sources of tetanus spores may be poor hygiene combined with poor wound management [9]. Despite being a vaccine-preventable disease, tetanus remains a public health challenge in developing countries like Uganda with high case-fatality rates ranging between 17.8% and 64% [10–11].

The first reported case of tetanus infection following SMC in Uganda occurred in July 2013 following a conventional surgical procedure with tetanus toxoid vaccination administered on the day of surgery. Six other tetanus cases were reported between August 2014 and July 2015 and were investigated by the MoH Safety Monitoring Team. These cases, and seven others from three African countries, were considered to be consistent with or indeterminately associated with circumcision [12]. The risk of tetanus was estimated at almost 35 times higher when an elastic collar compression device was used compared to conventional surgery [12]. Further investigations of the burden of tetanus infection in Uganda have been documented [11].

In Uganda, immunization of both adults and children is a mandate of the Ministry of Health through the Uganda National Expanded Program on Immunization (UNEPI). UNEPI is located in the department of National Communicable Disease Control (NCDC) within the Directorate of Clinical and Community Services. Tetanus is one of the immunizable diseases that UNEPI vaccinates against following a well-defined schedule as guided and recommended by the World Health Organization (WHO). In Uganda, MoH policy recommends tetanus toxoid (TT) vaccination for all women of childbearing age (15–45 years), with emphasis on pregnant women, especially during the antenatal care visits, and on school health programs [13]. Women of reproductive age are provided with the first dose at first contact with the health care worker, while the booster doses are provided as follows: 2^nd^ dose at four weeks after dose 1, 3^rd^ dose six months after the dose 2, 4^th^ dose one year after dose 3, and 5^th^ dose at one year after dose 4. Completion of all scheduled doses ensures life-long protection against tetanus infection. Although the tetanus vaccination for women is clearly laid, there is no vaccination policy for boys and men following infant immunization.

Lack of tetanus vaccination policy for males may compromise the quality of circumcision services, increase the risk of infection, and impact service uptake. Therefore, in 2015 WHO identified areas for which evidence was needed to make recommendations on improved SMC safety, including antibody titres prior to TT vaccination, antibody response to tetanus toxoid-containing vaccines (TTCV), and the correlation between antibody titres and prior tetanus vaccination [9]. In order to generate this evidence, we assessed the prevalence of TTCV and the immunological response following vaccination among men seeking circumcision services in a setting with a high burden of tetanus. This followed Uganda’s adoption of the WHO TT guidelines [9], which recommended two TTCV injections, 28 days apart, with the second TTCV administered on the day of circumcision.

## Materials and methods

### Study area

Thirteen SMC sites in five regions of the Uganda were selected to participate in the study: Rakai Health Science Program and Nkozi Hospital in Central Region; Mukono Hospital, Kojja Health Center (HC) IV, and Kayunga Hospital in Central-1 Region; Kabwohe HC IV, Munobwa Tea Estate Clinic, and Mbarara and Fort Portal Regional Referral Hospitals in Western Region; Busesa HC IV and Masfau and Princess Diana Hospitals in Eastern Region; and Gulu Regional Referral Hospital in Northern Region. All the sites provided SMC services through implementing partners working with the Ministry of Health. All the sites were assessed for eligibility based on availability of laboratory facilities to draw and process blood, store the serum, and transport samples for testing. The investigators, study coordinator, and the laboratory technologist trained field teams in research ethics and the study protocol.

### Recruitment of participants

All males aged 10 years or older seeking SMC services who were eligible for SMC were invited to participate in the study. Written informed consent for study participation was obtained from men aged 18 years and older and from parents or guardians of minors (aged 10 to 17 years). Minors also provided written informed assent in addition to parental/guardian consent. The study informed consents/assents were in addition to the routine consent/assent administered in the SMC program.

Clients were provided health -ion, voluntary HIV testing and counseling, and information on the benefits of circumcision, adverse events following circumcision, wound care and management, sanitation and hygiene, abstinence from intercourse for at least six weeks after circumcision and complete wound healing, and tetanus infection and vaccination. SMC teams at the study sites conducted data collection.

Participants completed an interview on social-demographic characteristics, knowledge of tetanus infection and its causes, and prior receipt of tetanus vaccination.

We consecutively enrolled consenting/assenting clients until the pre-determined sample size of 769 was achieved. Prior to administration of dose 1 of TTCV, venous blood was drawn from 256 clients randomly selected from all enrolling sites, who agreed to extra blood draws on days 14, 28, and 42 following enrollment.

On each follow-up visit after receipt of the TTCV, client’s identity was verified, and confirmed their willingness to continue participation in the study. Blood sample collection and processing used standard operating procedures. All samples were labeled with a pre-printed, computer-generated study identifier and visit number.

On the 28-day visit, after the blood sample was drawn, a second TTCV dose was administered before circumcision was performed as per MoH guidelines. A final blood draw was obtained on day 42.

### Sample size

The sample size estimates for the prevalence of protective tetanus antitoxin antibodies was 769 participants based on the following assumptions; 5% type-I error, 5% precision, 50% prevalence of protective antibodies and a design effect of 2.0. A total of 256 participants were estimated for the assessment of immunological response assuming 95% of TTCV-naïve clients would achieve a protective antitoxin concentration compared to 100% of those receiving a booster, over three follow-up visits, adjusting for individual correlation of 0.5 due to repeated observations and a loss to follow-up of 20%.

For this analysis, a total of 620 men with complete data on questionnaires and blood sample draw are presented, where 247 men have data on at least one follow-up.

Blood was stored in a cool box with ice and centrifuged within 12 hours of collection at the sites. Serum aliquots were labeled using the computer-generated identifier, and serum was stored at -20°C, until transportation in cool boxes with the ice packs to the testing laboratory at the Rakai Health Sciences Program in Kalisizo, Rakai. A field coordinator and quality control expert supported the study through regular site visits to review study operations, assure adherence to standard operating procedures, and restock sites with supplies. The investigators ensured adherence to the protocol through weekly visits to the sites and phone calls.

The Institutional Review Board of the School of Bio-medical Sciences at the College of Health Sciences, Makerere University, and the Uganda National Council of Science and Technology approved the study. We obtained permission from the President’s office for research, the respective district health office, and from the facility in-charges. All sites received tetanus toxoid vaccines from the district health office following the UNEPI guidelines.

### Laboratory testing

Anti-tetanus toxoid IgG antibody levels were determined using a commercially available indirect ELISA kit (EuroImmun AG, EI 2060–9601 G, Lubeck, Germany). Results were qualitatively interpreted as per kit insert using the categories: i) Insufficient immunity (<0.1 IU/mL) ii) Need Booster (0.1–0.5 IU/mL), iii) Booster needed 2–5 years (>0.5–1.1 IU/mL), iv) Booster needed 5–10 years (>1.1–5.0 IU/mL) or v) Booster needed 10 years (>5.0 IU/mL). Only one template was tested per day, and a 10% random sample of each template was re-assayed for quality control. All samples were screened in parallel for HIV-1/2 using two independent rapid diagnostic tests: the Determine HIV-1/2 (Alere Medical Company Limited, Chiba, Japan) and the HIV-1/2 Stat-Pak dipstick (HIV-1; Chembio Diagnostic Systems, Medford, NY). All concordant results were recorded as final. Discordant results were resolved using a tiebreaker test, the Uni-Gold HIV (HIV-1; Trinity Biotech, Bray, Ireland). The tests were performed and interpreted according to the manufacturer’s instructions. An experienced laboratory technologist blinded to the tetanus immunization status did all tests. A well-trained data manager prepared the laboratory data sets.

### Data management and statistical analysis

The data management team at the Rakai Health Sciences Program (RHSP) generated the computer identifications for samples and questionnaires. Data management teams at the RHSP, at the USAID ASSIST Project office in Kampala, and at the Makerere University School of Public Health captured questionnaire data electronically. All the questionnaire data were double entered using FoxPro version 9.0 (at the RHSP) and CSPro, cleaned and appended to form one dataset. Data were transferred into Stata version 14 for statistical analyses.

Descriptive analyses were conducted for the participants’ sociodemographic characteristics, knowledge about tetanus, prior receipt of tetanus vaccination, and presence of jiggers or current/recently healed wounds. For categorical variables, summary analyses were presented as percentages, and for continuous data, means (SD) and median (IQR) were generated. The sero-prevalence of antibodies against tetanus toxoid was determined as the proportion of clients with the following categories of the laboratory results; Need booster or booster needed 2 or more years divided by the number of clients with a test result at each study visit. The seroprevalence was also stratified by age, self-reported prior receipt of tetanus vaccination, and HIV status. The differences in IgG antibody seroprevalence by study visit, age, and HIV status was assessed by chi-square and Fisher’s exact tests. Statistical significance was based on a two-sided 5% type-I error.

The tetanus antibody concentrations (IU/mL) ranging from 0 to 5.1^+^ were analyzed using descriptive statistics (means, SD; median, IQR) and presented graphically using Spaghetti plots. In order to determine the rate of immunological response of clients following TTCV at days 0 and 28, a mixed effects model was used to estimate the rates of change in levels of antibody concentrations, with corresponding 95% confidence intervals.

## Results

A total of 620 clients had both questionnaire and laboratory data. The mean age of participants was 21.4 (SD 7.6) years, and nearly half (49.7%) had post-primary education. Sexual debut was reported by 53.4%, wherein 17.4% were currently married and 38.8% reported non-marital relationships. About two thirds (65.2%) responded affirmatively about knowledge of tetanus infection, and just over a half (56.6%) knew how tetanus was contracted. Self-reported receipt of tetanus toxoid-containing vaccine (TTCV) was low (22.8%). However, willingness to receive tetanus vaccination prior to circumcision was high (99.6%).

For the follow-up component, 256 randomly selected participants consented. The follow-up rate was high, with blood draws obtained from 237 (92.5%) on day 14, 213 (83.2%) on day 28, and 147 (57.4%) on day 42.

Table 1 shows the sero-prevalence of tetanus toxoid antibody categories at enrollment and by days since receipt of the TTCV. On day 0 (enrollment), 57% (354/620) had insufficient immunity, and 24.7% needed a booster dose as per the test kit insert. The percent of clients with insufficient immunity significantly declined to 7.3% within 14 days of receipt of the first TTCV dose, to 3.4% at day 28, and to zero at day 42 following a second TTCV dose administered on day 28.

**Table 1:** Sero-prevalence of tetanus antibody protection at enrollment.

	Days since tetanus toxoid vaccination			
	0	14	28	42
	N (%)	N (%)	N (%)	N (%)
Total, N	620 (100)	237 (100)	213 (100)	147 (100)
**Interpretation: Laboratory results**				
Insufficient immunity	354 (**57.0**)	17(**7.3**)	8 (**3.8**)	0
Need booster	153 (24.7)	18 (7.6)	9(4.2)	0
Booster needed 2–5 years	53 (8.5)	8 (3.4)	14 (6.6)	6 (4.1)
Booster needed 5–10 years	39(6.3)	35 (14.8)	44 (20.7)	38 (25.9)
Booster needed over 10 years	21 (3.4)	159 (67.1)	138 (64.8)	103 (70.1)

Table 2 shows the prevalence of protective tetanus toxoid antibodies by age and HIV status and days since receipt of TTCV vaccination. At day 0, the prevalence of protective antibodies was low and similar across age groups. At follow-up, the prevalence of protective antibodies increased up to 100% by day 42. Among the HIV-negative clients, the proportion with insufficient immunity declined rapidly after TTCV, whereas among the HIV-infected participants, insufficient immunity persisted for the duration of observation. On day 14, insufficient immunity was 5.0% among HIV-negatives compared to 54.5% among HIV-positives (p<0.001), and at day 28 the differential protection was 1.4% in the HIV-negatives and 62.5% in the HIV-infected (p<0.001). However, irrespective of HIV status, all clients were fully protected by day 42 following receipt of the two TTCVs, but the number of HIV-infected participants was small.

**Table 2:** Sero-prevalence of tetanus antibody protection by age and HIV status at enrollment.

	Days since tetanus toxoid vaccination			
	0	14	28	42
Total, N	N (%)	N (%)	N (%)	N (%)
Overall	620 (42.6)	237 (92.8)	213 (96.1)	147 (100.0)
Age (years)*				
10–14	88 (45.5)	49 (95.9)	44(100.0)	30(100.0)
15–19	216 (44.0)	87(96.6)	74(100.0)	60(100.0)
20–24	156 (39.7)	48(91.7)	46(95.7)	29(100.0)
25–29	78 (42.3)	22(90.9)	18(88.9)	13(100.0)
30–34	32(43.8)	12(100.0)	11(100.0)	8(100.0)
35+	40 (40.0)	17(64.7)	14(71.4)	7(100.0)
**HIV-negative**				
Total, N	585 (%)	220(%)	199 (%)	138 (%)
**Laboratory results**				
Insufficient immunity	331 (56.6)	11 (5.0)	3 (1.4)	
Need Booster	145(24.8)	16 (7.3)	8 (4.0)	
Booster needed 2+ years	109 (18.6)	193 (87.7)	188 (94.5)	138 (100)
**HIV-positive**				
Total, N	19 (%)	11(%)	8 (%)	4
**Laboratory results**				
Insufficient immunity	12(63.2)	6 (54.5)	5 (62.5)	-
Need booster	6 (31.6)	-	1 (12.5)	-
Booster needed 2+ years	1 (5.3)	5 (45.5)	2 (25.0)	4 (100)

* 10 respondents had missing age.

Table 3 shows the estimated mean and median tetanus toxoid antibody concentrations. The overall median (IQR) was 0.06 (0.03, 0.25) IU/mL on day 0 but significantly increased to 5.1 (2.7, 5.1) IU/mL and 5.1 (3.6, 5.1) IU/mL at days 14 and 28, respectively, following the first TTCV (p<0.001). The median maintained the maximum available reading of 5.1 (4.6, 5.1) on day 42 after a booster TTCV on day 28. Similar increases over time since the first TTCV were observed by age and HIV status. Over time, variation in TTCV antibody concentrations became smaller, suggesting that all participants attained the similar antibody at the end of the follow-up period. For clients aged 35 years and older, the median antibody concentration was significantly lower than that of the younger clients at all visits after day 0 (p<0.01).

**Table 3:** Estimated mean (SD) and median (IQR) of antibody concentration.

	Days since tetanus toxoid vaccination (IU/ml)							
	0		14		28		42	
**Total, N**	620		237		213		147	
Mean		0.44		3.86		4.05		4.48
SD		1.1		2.0		1.8		1.2
Median		0.06		5.1		5.1		5.1
IQR		0.03,0.25		2.7, 5.1		3.6, 5.1		4.6, 5.1
*Age (years)	n	**Median (IQR)**	n	**Median (IQR)**	n	**Median (IQR)**	n	**Median (IQR)**
10–14	88	0.06 (0.04, 0.195)	49	5.1 (3.91, 5.1)	44	5.1 (5.0, 5.1)	30	5.1 (5.1, 5.1)
15–19	216	0.06 (0.03, 0.195)	87	5.1 (4.99, 5.1)	74	5.1 (4.6, 5.1)	60	5.1 (4.9, 5.1)
20–24	156	0.05 (0.03, 0.285)	48	5.1 (0.74, 5.1)	46	5.1 (0.99, 5.1)	29	5.1 (4.0, 5.1)
25–29	78	0.06 (0.02, 0.290)	22	5.1 (0.69, 5.1)	18	5.1 (4.8, 5.1)	13	5.1 (5.1, 5.1)
30–34	32	0.06 (0.03, 0.925)	12	4.2 (2.2, 5.1)	11	3.3 (1.4, 5.1)	8	4.8 (3.3, 5.1)
35+	40	0.05 (0.03, 0.175)	17	0.89 (0.02, 5.1)	14	1.2 (0.09, 5.1)	7	3.2(1.6, 5.1)
*HIV status								
Negative	585	0.06(0.03, 0.26)	220	5.1(3.3, 5.1)	199	5.1(4.2, 5.1)	138	5.1(4.8, 5.1)
Positive	19	0.05(0.01, 0.15)	11	0.02(0.02, 4.21)	8	0.05(0.04, 0.58)	4	1.3 (0.97, 3.1)

Note

* Sample sizes per study visit may not add up due to missing data on HIV status or age

At enrollment, the prevalence of HIV was highest among clients 35 years or older (20%, 8/40). The median antibody titres of HIV-positive subjects were significantly lower than for the HIV-negative subjects at all follow-up visits (p<0.001), but similar at enrollment (p = 0.0721).

### Sub-analysis: Individual level

A total of 247 HIV-negative clients had at least one follow-up study visit, where 49.4% (122/247) had all the four visits and 26.7% (66/247) and 23.9% (59/247) had three and two visits, respectively.

**Fig 1:**
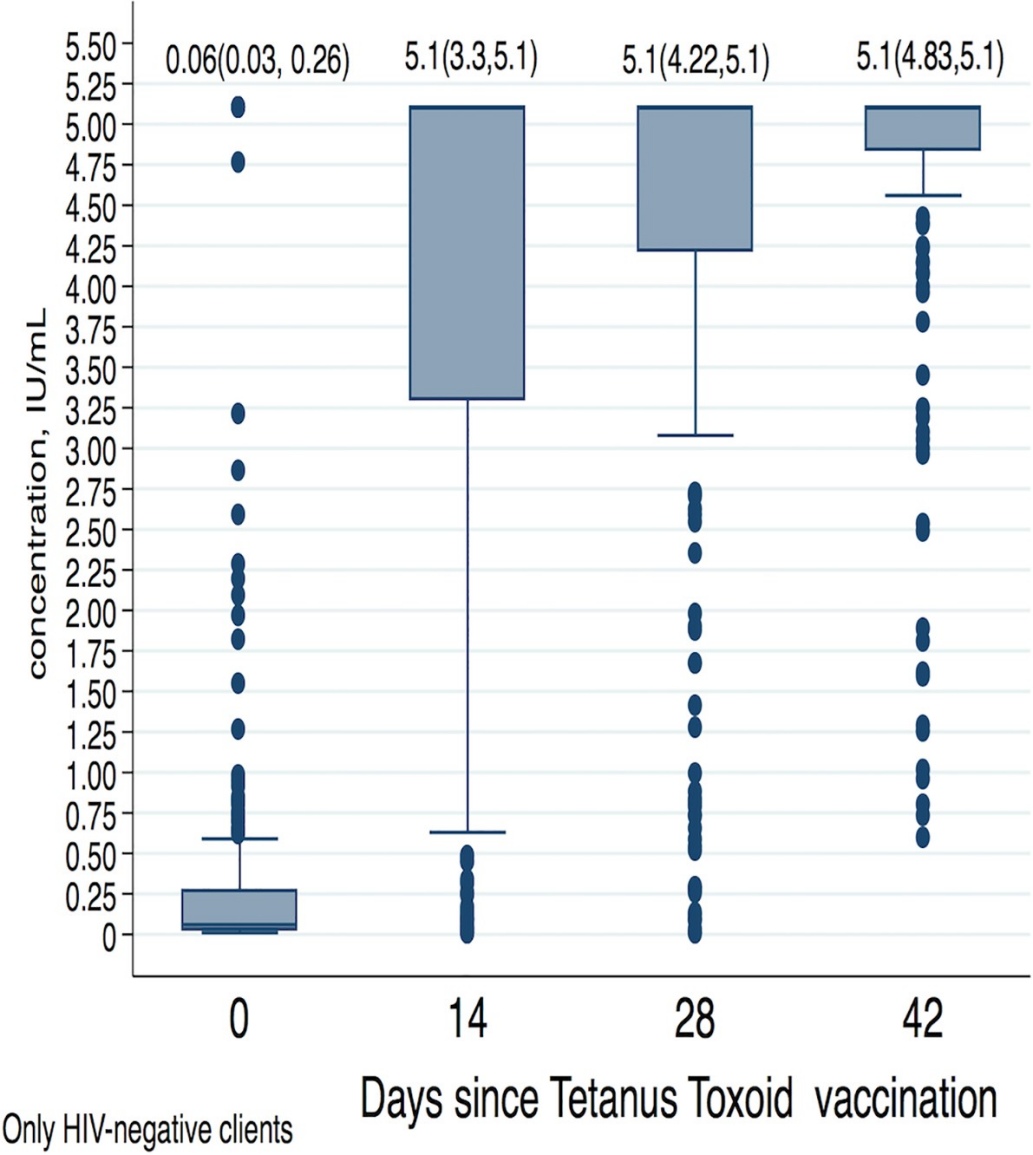
Whisker box plot: Median antibody concentration over days since first vaccination following enrolment into study.

**Fig 2:**
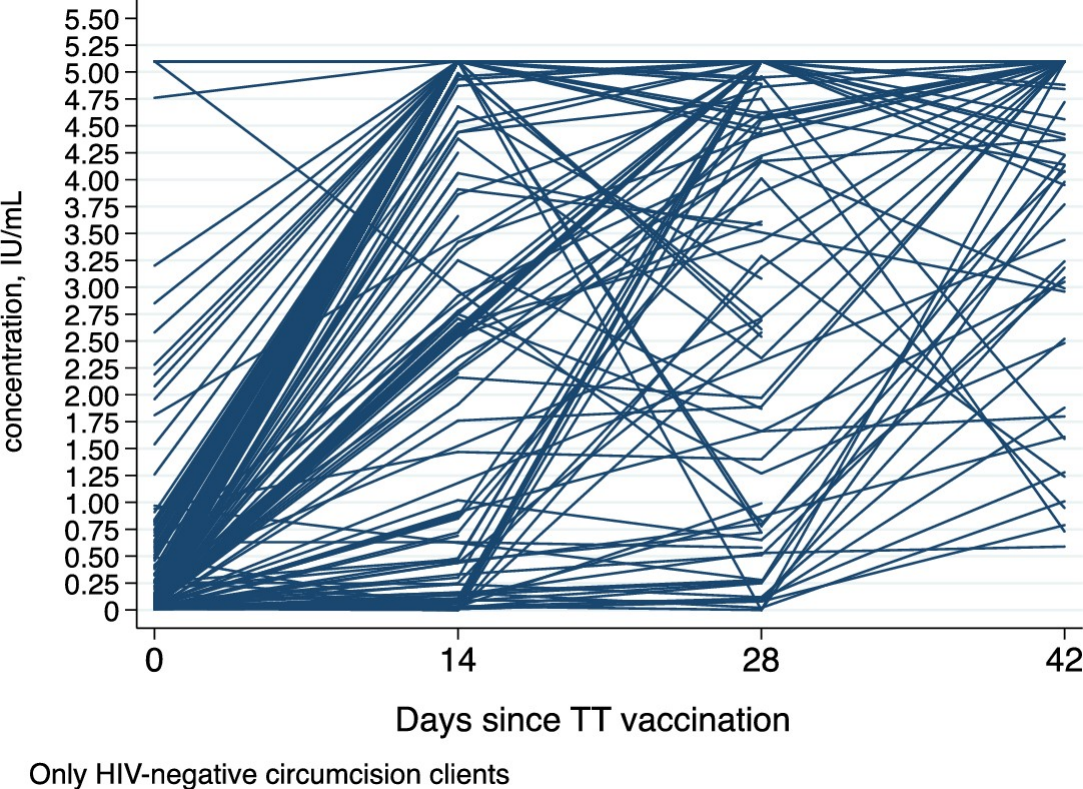
Spaghetti plot: Observed antibody concentration trajectories for each individual participant.

Fig 1 shows the Whisker Boxplot of antibody concentration for only the HIV negative participants by day since receipt of the first anti-tetanus toxoid vaccination in the study. The median (Inter-quartile range) of anti-tetanus toxoid concentration is indicated on top of each boxplot. The median (IQR) antibody concentration rapidly increases from 0.06(0.03,0.26) at day 0 to 5.1 (3.3, 5.1) at day 14. Also three-quarters (75%) of the participants had antibody concentration of 0.03 or more on day 0, and by day 14 the concentration had rapidly increased to 3.3 and to 4.2 on day 28 prior to receipt to a second tetanus toxoid vaccination. Fig 2 shows a Spaghetti plot where an increase in each individual participant’s antibody concentration was observed. This plot suggests trajectories that are nonlinear and which increase rapidly from day 0 to day 14 and then slow down. The plot also shows variation in individual clients’ antibody concentrations on day 0 and a possible variation in the trajectories. We therefore applied a mixed effects model with a quadratic term for the day since vaccination in the model and a covariance structure assumed as unstructured. The model also adjusted for client’s age and category of antibody concentration at enrollment. The coefficient for days since vaccination, a measure of rate of change, was 0.27 (95% CI: 0.25, 0.29), and the quadratic term for days since vaccination was -0.004 (95% CI: -0.0047, -0.0038); both were statistically significant, p<0.001.

## Discussion

The study found that 57% of men attending SMC services had insufficient immunity to tetanus. The prevalence of protective tetanus toxoid antibodies increased rapidly following TTCV by day 14. The finding of sero-prevalence of protective tetanus antibody levels of 43% is consistent with DPT3 coverage in Uganda of 50% prior to 2000, although the latter increased to 85% in 2007 [12]. Moreover, sero-prevalence at enrollment was similar across all ages. This finding suggests that DPT3 coverage has not significantly improved over time, especially in the rural settings where most of the circumcision clients reside [12].

Tetanus infection in Uganda is still common with a two fold increase in the number of female cases aged 5 or more old were observed from 755 in 2011 to 1665 in 2014; similarly the number of males aged 5 years or older increased from 1007 to 1311 in the same period [11], and wounds due to trauma have been reported, especially among farmers [14]. Road traffic injuries are also common [15–16], as are Jigger infestations (Tungiasis) [17]. Therefore, the risk of current or recently healed wounds and jigger infestations among SMC clients remains a public health challenge which needs to be addressed with better tetanus toxoid vaccination coverage and wound care to minimize the risk of tetanus [18]

This study suggests a rapid immunological response within the first 14 days following TTCV when the majority of clients attained protective antibody levels. Previous studies have only provided data at day 28 as the first follow-up visit, and so it was not empirically possible to know how much earlier the immunological response occurs [19].

The low sero-prevalence of tetanus toxoid antibody concentration and poor immune response in HIV-positive or older (35+ years) clients is consistent with previous studies [20]. The poor immune response in the older age may be confounded by the high HIV prevalence (20%) in this age group. The sample size in this age group was too small to make more meaningful analysis. The poor immunologic response to TTCV among HIV-positive men may be due to depletion of B lymphocytes and impaired humoral immunity in immune-compromised individuals [21–23]. These findings suggest the importance of HIV testing and counseling for clients seeking circumcision [13].

The study was conducted within routine SMC services, and findings are likely to be generalizable to other circumcision programs. However, the study population of clients seeking circumcision services is likely to be self-selected, in good health, and may not be representative of the general male population.

Baseline protective antitoxin levels depend on the coverage of prior vaccination programs, which may vary across different regions, but the study was not powered to detect regional differences.

## Conclusion

There remains a significant risk of tetanus among men seeking circumcision in Uganda due to insufficient immunity against tetanus infection (57%). Tetanus toxoid immunization results in a rapid immunological response, providing almost universal protection by day 14 after immunization.

## Recommendations

Circumcision may be conducted 14 days following TTCV, and clients should be encouraged to return for a second immunization after their last post-operative visit. However, screening for HIV and older age is needed since there is a slow or delayed response to TTCV. In the long-term, mass tetanus vaccination campaigns should be conducted, and men should be offered a TTCV booster at time of first contact for SMC if documentation of participation in mass vaccination is available.

## References

[1] Auvert B, Taljaard D, Lagarde E, Sobngwi-Tambekou J, Sitta R, Puren A. Randomized, controlled intervention trial of male circumcision for reduction of HIV infection risk: the ANRS 1265 Trial. *PLoS Med*. 2005 11;2(11):e298 Epub 2005 Oct 25. 10.1371/journal.pmed.0020298 16231970 PMC1262556

[2] Bailey RC, Moses S, Parker CB, Agot K, Maclean I, Krieger JN et al Malecircumcision for HIV prevention in young men in Kisumu, Kenya: a randomised controlled trial *Lancet*. 2007 2 24;369(9562):643–56. 10.1016/S0140-6736(07)60312-2 17321310

[3] Gray RH, Kigozi G, Serwadda D, Makumbi F, Watya S, Nalugoda F, et al Male circumcision for HIV prevention in men in Rakai, Uganda: a randomised trial. *Lancet*. 2007; 369(9562):657–666. 10.1016/S0140-6736(07)60313-4 17321311

[4] UPHIA 2016 Uganda Population-Based HIV Impact Assessment (UPHIA) 2016–2017. Available online from: www.afro.who.int/sites/default/files/201708/UPHIA%20Uganda%20factsheet.pdf

[5] Ministry of Health (MoH). Ministry of Health Safe Male Circumcision (SMC) Death Audit Report Kampla, Uganda: Ministry Of Health, Safety Monitoring Team 11 2014.

[6] Kigozi G, Musoke R, Watya S, Kighoma N, Nkale J, Nakafeero M, et al The safety and acceptance of the PrePex device for non-surgical adult male circumcision in Rakai, Uganda. A non-randomized observational study. *PLoS One*. 2014 8 21;9(8):e100008 10.1371/journal.pone.0100008 25144194 PMC4140666

[7] Kankaka EN, Murungi T, Kigozi G, Makumbi F, Nabukalu D, Watya S, et al Randomized trial of early infant circumcision performed by clinical officers and registered nurse midwives using the Mogen clamp in Rakai, Uganda. *BJU Int*. 2017 1;119(1):164–170. 10.1111/bju.13589 27597563

[8] Soumaré M, Seydi M, Dia NM, Diop SA, N'dour CT, Diouf L, Diop BM, Sow PS. Post-circumcision tetanus in Dakar, Senegal Bull Soc Pathol Exot. 2008 2;101(1):54–7 18432010

[9] WHO informal consultation on tetanus and voluntary medical male circumcision. 2015. Geneva, Switzerland: World Health Organization.

[10] Oshinaike OO, Ojelabi OO, Ogbera AO, Ojo OO, Ajose FA, & NU O. (2012). Improving case fatality rate of adult tetanus in urban Nigeria: focus on better facilities of care. *Trop Doct*. 10.1258/td.2012.120250 23117957

[11] Nanteza B, Galukande M, Aceng J, Musinguzi J, Opio A, Mbonye AK, et al The burden of tetanus in Uganda. *Springerplus*. 2016 6 10;5(1):705 10.1186/s40064-016-2309-z eCollection 2016. 27350934 PMC4902800

[12] WHO and UNICEF estimates of immunization coverage: 2016 revision. Available online from: www.who.int/immunization/monitoring_surveillance/data/uga.pdf downloaded June 29, 2018.

[13] Ministry of Health (MoH). National Guidelines on Management of Common Conditions. 2010.

[14] Lunner-Kolstrup C, Ssali TK. Awareness and Need for Knowledge of Health and Safety among Dairy Farmers Interviewed in Uganda. *Front Public Health*. 2016 6 28;4:137 10.3389/fpubh.2016.00137 eCollection 2016 27446901 PMC4923150

[15] Galukande M, Jombwe J, Fualal J, A, Gakwaya A Boda-boda Injuries a Health Problem and a Burden of Disease in Uganda: a Tertiary Hospital Survey. *East Cent*. *Afr*. *J*. *Surg* Vol 14 No2 2009.

[16] Tumwesigye NM, Atuyambe LM, Kobusingye OK. Factors Associated with Injuries among Commercial Motorcyclists: Evidence from a Matched Case Control Study in Kampala City, Uganda. *PLoS One*. 2016 2 26;11(2):e0148511 10.1371/journal.pone.0148511 eCollection 2016 26918871 PMC4769300

[17] Wafula ST, Ssemugabo C, Namuhani N, Musoke D, Ssempebwa J, Halage AA. Prevalence and risk factors associated with tungiasis in Mayuge district, Eastern Uganda. Pan Afr Med J. 2016 5 24;24:77 10.11604/pamj.2016.24.77.8916 eCollection 2016. 27642416 PMC5012786

[18] WHO current recommendations for treatment of tetanus during humanitarian emergencies. 2010. Geneva, Switzerland: World Health Organization.

[19] Shohat T, Marva E, Sivan Y, Lerman I, Mates A, Cohen A. Immunologic Response to a Single Dose of Tetanus Toxoid in Older People. *J Am Geriatr Soc*. 2000 8;48(8):949–51 10968300 10.1111/j.1532-5415.2000.tb06893.x

[20] Choi JH, Choo EJ, Huh A, Choi SM, Eom JS, Lee JS, et al Immunogenicity and Safety of Diphtheria-tetanus Vaccine in Adults. *Infectious Diseases*, *Microbiology & Parasitology*. *J Korean Med Sci*. 2010 12;25(12):1727–32. 10.3346/jkms.2010.25.12.1727 Epub 2010 Nov 24. 21165286 PMC2995225

[21] Alsina L, Noguera-Julian A, Fortuny C. Impaired cellular immune response to tetanus toxoid but not to cytomegalovirus in effectively HAART-treated HIV-infected children. *Vaccine*. 2013 5 7;31(20):2417–9. 10.1016/j.vaccine.2013.03.035 Epub 2013 Apr 5. 23562610

[22] Ching N, Deville JG, Nielsen KA, Ank B, Wei LS, Sim MS et al Cellular and humoral immune responses to a tetanus toxoid booster in perinatally HIV-1-infectedchildren and adolescents receiving highly active antiretroviral therapy (HAART). *Eur J Pediatr*. 2007 1;166(1):51–6. Epub 2006 Jul 26. 10.1007/s00431-006-0184-2 16868780

[23] Rosenblatt HM, Song LY, Nachman SA, Stanley KE, Krogstad PA, Johnson GM et al Tetanus immunity after diphtheria, tetanus toxoids,and acellular pertussis vaccination in childrenwith clinically stable HIV infection. *J Allergy Clin Immunol*. 2005 9;116(3):698–703 10.1016/j.jaci.2005.05.016 16159645

